# Integrative Yoga and Ayurvedic Approach to Oligoasthenozoospermia: A Holistic Case Study on Fertility Enhancement

**DOI:** 10.7759/cureus.55566

**Published:** 2024-03-05

**Authors:** Bhagwat Gade, Jarul Shrivastava, Namrata Choudhary, Gauri Gajabe, Shilpa Dutta, Ritesh Jadhav, Akash More

**Affiliations:** 1 Clinical Embryology, Datta Meghe Institute of Higher Education and Research, Wardha, IND; 2 Anatomy, Datta Meghe Institute of Higher Education and Research, Wardha, IND

**Keywords:** test tube baby, male infertility, ayurveda, yoga, oligoasthnozoospermia

## Abstract

Infertility is the failure to conceive after one or more years of regular, unprotected life for a fertile female. Around 45% of males are responsible for infertility worldwide. Research shows that nearly 50% of infertility in India is related to male reproductive factors or diseases. The male-carrying pathology in semen production includes low sperm count, volume, motility, abnormal forms, and sperm functional tests. This case presents a 31-year-old male with complaints of wanting issues after a complete year of regular, unprotected intercourse. He had undergone all the routine diagnostic investigations on his wife, which reported no issues and recorded regular ovulatory cycles with patent tubes. Then, progressing in the diagnosis, a semen analysis revealed a semen volume of 2 mL, a sperm concentration of 4 million/mL, progressive motility of 8%, non-progressive motility of 3%, and immotile sperm of 89%, with normal sperm morphology. Based on clinical examination, semen analysis, and investigation, the case was diagnosed as oligoasthenozoospermia. Oligozoospermia means low sperm count, and asthenozoospermia means low sperm motility. Oligoasthenozoospermia can be correlated to the Shukra Kshaya Lakshanas mentioned in Ashta Shukra Dushti. There is no satisfactory treatment in modern medicine for these conditions. Yoga and Ayurvedic intervention are the better options for these conditions. This case report focuses on the management of oligoasthenozoospermia through yoga and Ayurvedic medicines, Youvanamrit Vati and Shilajitrasayan Vati, given to the patient for four months.

## Introduction

The inability of a couple to conceive after 12 months or more of regular, unprotected sexual activity is known as infertility [1]. It is frequently brought on by problems, including hormone imbalances or reproductive diseases, that affect one or both partners. A thorough medical evaluation is necessary for diagnosis, which may call for specialized care like assisted reproductive technologies (ART) [2,3]. Infertility impacts 8% to 12% of couples worldwide, with male infertility serving as a primary or contributing factor in about 50% of cases. This condition arises from various etiologies, including genetic, hormonal, and environmental factors, leading to impaired sperm production, function, or transport. Diagnostic evaluation entails semen analysis, hormonal assays, and genetic testing to elucidate underlying causes [3,4].

According to the WHO, global infertility affects 60-80 million couples. In India, 10-15% of women of reproductive age and 25% of men are infertile [5]. Factors contributing to infertility include genetic predispositions, infections, hormonal imbalances, and lifestyle choices. Treatment options range from medication and surgery to ART such as in-vitro fertilization (IVF) and intrauterine insemination (IUI). Early diagnosis and intervention are crucial for managing infertility [6]. A blockage that stops sperm from being delivered, low sperm production, or poor sperm function are all signs. Oligoasthenozoospermia is a sperm disease that co-occurs with two other conditions - asthenozoospermia (abnormal sperm motility, with more than 60% of sperm being immotile or unable to move in a straight path) and oligozoospermia (low sperm count, below 15 million/mL) [7]. Vajikarana is a specialized branch of Ayurveda dealing with Shukradushti. Ksheena Shukra is included in one of the varieties of Dosha that are vitiated; the quality and quantity of the Shukra alter and result in Shukradushti, especially Ksheena Shukra [8,9]. Yoga and Vajeekaranadravya, traditional Ayurvedic formulations, have shown efficacy in increasing sperm count and motility. Vajeekaranadravya, composed of specific herbs and minerals, enhances reproductive health through its antioxidant and hormonal-balancing properties. Yoga complements this by reducing stress, a significant factor in male infertility, thus optimizing sperm parameters [10].

## Case presentation

Patient information

The six-year-old married couple at the center of this case study obtained treatment at Acharya Vinoba Bhave Rural Hospital after a five-year unsuccessful attempt at pregnancy. The partner, who was 31 years old, was a farmer by trade, working in the agricultural sector. They had difficulties becoming pregnant, even though they had regular sexual intercourse for six years without using birth control. The female partner had a normal anti-Müllerian hormone (AMH) level, i.e., 4.38 ng/mL, and a good antral follicle count, i.e., 12. She had regular menstrual cycles and a normal bleeding pattern, according to a thorough assessment of the situation. The male patient was requested to self-masturbate to collect semen for a semen analysis after providing a complete medical history. The 31-year-old male partner's semen examination showed that his semen volume was 2 mL, his sperm concentration was 4 million/mL, his quick progressive motility was 8%, his non-progressive motility was 3%, and he was immotile at 89%, with 1-2 pus cells/high power field (hpf) present.

Furthermore, no red blood cells or epithelial cells were found. The sample had a longer liquefaction time and normal sperm morphology. A physical checkup revealed no abnormalities. Oligoasthnozoospermia, also known as Ksheena Shukra in Ayurvedic medicine, was diagnosed by the patient based on the results of the laboratory tests and the clinical history.

Medical history

The patient had been married for six years and had engaged in unprotected sexual activities without the use of birth control. After undergoing an endocrinological and clinical assessment, the partner of the patient was healthy. For the past five years, she had engaged in frequent, unprotected sexual activity, even on the 12th to 18th day of her menstrual cycle, but she was never able to conceive. He worked on a farm as an employee. The patient's medical history did not mention any long-term attenuating diseases or illnesses, such as hydrocele, tuberculosis, mumps, or others. He had never taken gonadotoxic drugs and had no prior surgical history, including no vasectomy or herniorrhaphy. There is no history of early ejaculation or erectile problems in the patient. The only differences were in the male patient's motility, morphology, and sperm count. The patient was advised to have his semen examined after observing the recommended three days of abstinence. The semen examination findings before starting the medicine revealed a low sperm count of 4 million cells/mL, with quick progressive motility of 8% and non-progressive motility of 3% immotile at 89%; 1-2 pus cells/hpf were present. Furthermore, no red blood cells or epithelial cells were found.

Other Details of the Patient

Based on the information provided, the 31-year-old farmer had a normal general physical examination and testicular observation, as mentioned in Table 1. No abnormalities are noted in the reproductive system, including testis size consistency, vas deferens, epididymis, varicocele, scrotal swelling, inguinal examination, or rectal examination (prostate).

**Table 1:** Physical examination of testis cm, centimeter

Testis physical examination	Right	Left
Testis size consistency	3.8 × 2.5 × 2 cm rubbery	3 × 2 × 1.5 cm rubbery
Vas deferens	Soft	Soft
Epididymis	Felt smooth	Felt smooth
Varicocele	Nil	Nil
Scrotal swelling	Nil	Nil
Inguinal examination	No any scar	No any scar
Rectal examination (prostate)	Normal	Normal

Treatment

While modern medical approaches offer solutions, Ayurveda, the ancient Indian system of medicine, provides holistic and comprehensive strategies for addressing male infertility.

Proper Dietary Management

Ayurveda emphasizes the importance of diet in promoting reproductive health. Patients are advised to consume a balanced diet consisting of milk, vegetables, salads, cow ghee, dairy products, and coconut oil. Additionally, specific dietary recommendations include incorporating old jaggery, castor oil, sharkara, wheat, bananas, and red rice into daily meals. It is essential to limit the intake of salty, pungent, bitter, and excessively flavored foods, as these may negatively affect reproductive function.

Lifestyle Changes

Adopting lifestyle modifications plays a crucial role in managing male infertility according to Ayurvedic principles. Patients are counseled to minimize stress, anxiety, excessive exercise, and sexual activity, as well as refrain from suppressing natural urges (sleep, thirst, hunger, etc.). Substance abuse, particularly alcohol consumption, is discouraged due to its adverse effects on liver function and estrogen levels, which can impair sperm generation. Furthermore, recommendations include avoiding hot water baths, tight undergarments, long bike rides, and hot/spicy foods. Prioritizing adequate sleep, regular exercise, avoiding fast food, quitting smoking, and ensuring sufficient vitamin D intake are also emphasized.

Yoga Exercises

Ayurveda advocates the incorporation of specific yoga postures and practices to enhance reproductive health and increase sperm count. Regular yoga sessions facilitate stress reduction and improve overall well-being, positively impacting reproductive organs. Some beneficial yoga poses include Setu Bandhasana (bridge pose), Agnisar Kriya (fire-purification technique), Halasana (plow pose), Dhanurasana (bow pose), Ardha Matsyendrasana (half spinal twist pose), Padmasana (lotus pose), and Surya Namaskara (sun salutation). Additionally, Pranayama (breathing exercises) and meditation are recommended to promote relaxation and enhance the mind-body connection, contributing to improved fertility outcomes.

In conclusion, Ayurveda offers a comprehensive approach to addressing male infertility, encompassing dietary modifications, lifestyle adjustments, and specific yoga practices. By adopting these holistic interventions, individuals can optimize reproductive health, increase sperm count, and enhance overall well-being, thereby improving their chances of conception and achieving parenthood.

Intervention details of yoga are mentioned in Table 2.

**Table 2:** Type of yoga intervention and its duration [11]

Sr. no.	Yogic practices	Time duration
1	Surya Namaskara	5-6 min
2	Meditation	5-6 min
3	Pranayama/Kapalbhati	5-6 min
4	Padmasana	5-6 min
5	Setu Bandhasana	5-6 min
6	Tadasana	5-6 min
7	Dhanurasana	5-6 min
8	Uttanpadasana	5-6 min
9	Ashwini Mudra	5-6 min
10	Bhujangasana	5-6 min
11	Sarvangasana	5-6 min

Ayurvedic intervention

The patient was treated with Youvanamrit Vati (Divya Pharmacy), one tab (125 mg each), Shilajitrasayan Vati tablet (Divya Pharmacy), one tablet (500 mg each) twice a day with lukewarm water after meals, along with Shuddh Konch Beej Churna (100 g) - 3.5 g twice a day with milk. The treatment was continued for four months.

Composition of Youvanamrit Vati and Shilajitrasayan Vati

Youvanamrit Vati: Ashwagandha (*Withania somnifera*) (12 mg), Shuddha Kaunch (*Mucuna pruriens*) (12 mg), Bala (*Sida cordifolia*) (12 mg), Shatavari (*Asparagus racemosus*) (8.25 mg), Mush (*Chlorophytum arundinaceum*) (12 mg), Jaiphal (*Myristica fragrans*) (12 mg), Javitri (*Myristica fragrans*) (17.5 mg), Shuddha Kuchla (*Strychnos nux-vomica*) (2 mg), Akarkara (*Anacyclus pyrethrum*) (12 mg), Babul (*Acacia arabica*) (1.75 mg), *Swarna bhasma* (0.25 mg), Praval Pishti (0.25 mg), Vang Bhasha (5.5 mg), Makardhwaj Shuddha Shilajit (5.5 mg) processed with Paan Swaras excipient gum acacia, Aerosil, and Talcum quantum satis (QS).

Shilajitrasayan Vati: Ashwagandha (*Withania somnifera*), Bhoomi Amla, Harad (*Terminalia chebula*), Bahela (*Terminalia bellerica*), Amla (*Emblica officinalis*), and aqueous extract of Shilajit Shuddha.

Semen Analysis and Explanation

The semen analysis was conducted. There were 10 million sperm/mL, with 10% showing progressive motility, 4% with non-progressive motility, and 86% with immotile sperm. The sample exhibited higher viscosity and longer liquefaction times but had normal sperm morphology. Oligoasthenozoospermia was the impression left on the patient, who felt less hot and enthusiastic than before therapy.

A second semen analysis was performed, revealing 10 million sperm/mL, with 14% showing progressive motility, 7% with non-progressive motility, and 79% with immotile sperm. Similar to the previous sample, it displayed higher viscosity and longer liquefaction times but had normal sperm morphology, leaving an oligo asthenozoospermia impression. Details of semen analysis are mentioned in Table 3.

**Table 3:** Semen analysis performed before intervention, during intervention, and post-intervention mL, milliliter; min, minutes; hpf,

Date	First semen analysis	Second semen analysis	Third semen analysis
Method	Masturbation	Masturbation	Masturbation
Abstinence	Three days	Three days	Three days
Location of collection	Hospital	Hospital	Hospital
pH	7.2	7.2	7.2
Macroscopic examination			
Volume	2 mL	2.5 mL	2.5 mL
Color	Opaque grey	Opaque grey	Opaque grey
Odor	Seminal	Seminal	Seminal
Viscosity	Liquefied	Liquefied	Liquefied
Liquefaction time	15 min	15 min	15 min
Microscopic examination			
Sperm concentration	4 million cells/mL	10 million cells/mL	10 million cells/mL
Sperm motility			
Progressive motility	08%	10%	14%
Non-progressive motility	03%	4%	7%
Immotile	89%	86%	79%
Abnormal forms	96%	94%	90%
Other abnormalities			
Pus cells	1-2/hpf	1-2/hpf	Absent
Red blood cells	Absent	Absent	Absent
Epithelial cells	Absent	Absent	Absent
Impression	Oligoasthenoteraozoospermia	Oligoasthenozoospermia	Oligoasthenozoospermia

Follow-up

The female patient was advised to observe minimal physical exertion over the subsequent two weeks due to the critical nature of self-care in mitigating the risk of miscarriage. Additionally, she was prescribed multivitamins to bolster pregnancy outcomes. After this period, the patient returned to the hospital for a follow-up appointment, where a blood test was conducted to measure the serum β-human chorionic gonadotropin (hCG) levels at our laboratory in Sawangi, Maharashtra. The obtained β-hCG value of 1,100 mIU/mL confirmed a positive pregnancy outcome for the patient. Moreover, in men, the utilization of yoga and Ayurvedic treatments has been observed to enhance sperm concentration and motility, thereby positively impacting fertility outcomes.

## Discussion

This case study presents a 31-year-old male patient who presented with a chief complaint of ability to conceive after engaging in frequent, unprotected sexual intercourse for over five years. Subsequent investigations revealed a diagnosis of oligoasthenozoospermia, a condition characterized by low sperm count (oligozoospermia) and poor sperm motility (asthenozoospermia) [12]. Oligoasthnozoospermia is a leading cause of male infertility and can significantly impact a couple's ability to conceive [13].

In the pursuit of addressing his fertility concerns, the patient underwent a multifaceted treatment approach that included yoga and Virechana therapy. Yoga, a mind-body practice that combines physical postures, breathing exercises, and meditation, has been shown to have various health benefits, including stress reduction and improvement in reproductive health parameters [14]. Virechana therapy is a component of the Panchakarma cleansing procedures in Ayurveda, a traditional system of medicine originating from India. Virechana specifically involves therapeutic purgation to eliminate toxins from the body and restore balance [15].

Following the implementation of yoga and Virechana therapy, the patient reported a significant improvement in his fertility status, culminating in a successful pregnancy. Subsequent semen analysis revealed notable enhancements across various seminal parameters, including an increase in sperm count, improvement in sperm motility, enhancement in sperm morphology, reduction in the presence of pus cells, and alleviation of premature ejaculation. The observed improvements in semen characteristics following the integrative approach of yoga and Virechana therapy underscore the potential benefits of complementary and alternative medicine modalities in addressing male infertility.

While conventional treatments for oligoasthenozoospermia typically involve assisted reproductive techniques, such as IVF or IUI, this case highlights the potential role of holistic interventions in enhancing fertility outcomes. However, it is essential to note that further research is warranted to elucidate the mechanisms underlying the observed improvements and to establish the efficacy and safety of such interventions in the management of male infertility.

## Conclusions

This case report demonstrates that a combined approach utilizing yoga and Virechana intervention yields notable enhancements in sexual function parameters and semen characteristics. Through yoga and Ayurvedic treatment, significant improvements were observed not only in sexual function but also in the quality and quantity of semen. This integrated approach underscores the potential synergy between traditional practices, such as yoga and Ayurveda, in addressing aspects of sexual health. These findings advocate for further exploration and integration of complementary therapies in the management of sexual dysfunction, offering holistic solutions that consider both physical and psychological aspects of well-being.
